# Negative effects on oral motor function after submandibular and parotid botulinum neurotoxin A injections for drooling in children with developmental disabilities

**DOI:** 10.1111/dmcn.16131

**Published:** 2024-10-24

**Authors:** Reva M. van Eck, Lynn B. Orriëns, Corinne P. A. Delsing, Frank J. A. van den Hoogen, Corrie E. Erasmus, Karen van Hulst

**Affiliations:** ^1^ Department of Paediatrics, Division of Paediatric Neurology Radboud University Medical Center, Donders Institute for Brain, Cognition and Behaviour, Amalia Children's Hospital Nijmegen the Netherlands; ^2^ Faculty of Medical Sciences Radboud University Medical Center Nijmegen the Netherlands; ^3^ Department of Otorhinolaryngology/Head and Neck Surgery Radboud University Medical Center Nijmegen the Netherlands; ^4^ Department of Rehabilitation Radboud University Medical Center, Donders Institute for Brain, Cognition and Behaviour, Amalia Children's Hospital Nijmegen the Netherlands

## Abstract

**Aim:**

To evaluate negative effects on oral motor function after concurrent submandibular and parotid (four‐gland) botulinum neurotoxin A (BoNT‐A) injections as a treatment for paediatric drooling.

**Method:**

This was a retrospective cohort study of 125 children (median age 7 years 7 months [interquartile range 4 years 5 months]) with developmental disabilities, including cerebral palsy, treated with four‐gland injections. Most children (90.4%) were previously exposed to submandibular injections. Frequency, severity, and duration of negative effects on oral motor function (i.e. saliva swallowing, eating, drinking, articulation) were evaluated and compared to a reference cohort treated with submandibular injections.

**Results:**

Negative effects on oral motor function were reported in 45 children (36.0%), predominantly manifesting as eating‐related problems (64.4%). Most negative effects (62.2%) were classified as mild and resolved within 4‐weeks post‐injunction (53.3%). Compared to the reference cohort, frequency (36.0% vs 33.0%) and duration (53.3% vs 53.6% resolving within 4 weeks) of negative effects were comparable, although problems were more often moderately severe (33.3% vs 10.1%).

**Interpretation:**

While negative effects on oral motor function were relatively common after four‐gland BoNT‐A injections, most problems were mild and resolved promptly. No substantial differences to a reference cohort treated with submandibular injections were observed, although further research should establish the generalizability of these findings in a treatment‐naive population. Nevertheless, when submandibular injections prove ineffective, clinicians can confidently consider four‐gland injections.

## Graphical abstract

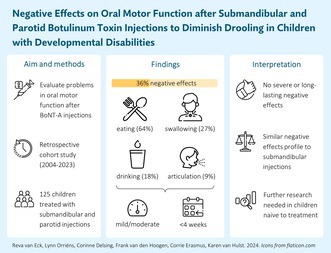

Graphical representation of the research objective, methodology, key findings, and implications.

Plain language summary: https://onlinelibrary.wiley.com/doi/10.1111/dmcn.16171

**Abbreviations**
DSFS: Drooling Severity and Frequency Scale
VAS: Visual Analogue Scale
vNRS: Verbal Numerical Rating Scale

**What this paper adds**

- Negative effects after four‐gland botulinum neurotoxin A (BoNT‐A) injections in children with developmental disabilities are common, yet mild and transient.
- Most frequent negative effects were related to eating difficulties and swallowing saliva.
- Subsequent four‐gland BoNT‐A injections (after previous submandibular injections) show similar negative effects to initial submandibular injections.
- Moderately severe negative effects were more frequent in the four‐gland treatment group.

While it is common for infants and toddlers to drool, this tendency typically diminishes through maturation of oral sensorimotor functions and is considered pathological after the age of 4 years.^1^ Nevertheless, persistent anterior (i.e. visible) drooling remains prevalent in children with cerebral palsy (CP) or other developmental disabilities,^2^ resulting in a variety of practical, physical, and social–emotional consequences that impact daily life and care.^1^, ^3^, ^4^ Moreover, some children with developmental disabilities experience posterior drooling,^5^ where saliva uncontrollably flows into the pharynx rather than spilling from the mouth, posing an increased risk of respiratory infections and significantly impacting their quality of life.^1^ This emphasizes the imperative for timely and effective treatment to address drooling in these children.

Intraglandular injections of botulinum neurotoxin A (BoNT‐A) are regarded as an effective treatment choice for drooling,^6^ alongside conservative treatment options, anticholinergic medications, or surgical interventions.^7^ BoNT‐A works by blocking the release of acetylcholine at the neuromuscular junction within the salivary glands, thereby decreasing saliva production.^6^ Nevertheless, no consensus has been reached on the most suitable major salivary glands to target (i.e. the submandibular glands, the parotid glands, or both). Whereas the submandibular glands produce the majority of resting saliva, which has a seromucous consistency, the parotid glands substantially increase their production of serous saliva when stimulated (e.g. during the chewing of food).^8^ To address this, our saliva control team at the Radboudumc Amalia Children's Hospital adopted a stepped care approach for children treated with BoNT‐A. The initial injections target the submandibular glands to diminish resting saliva production. If the response is deemed insufficient—after a shared decision‐making process involving healthcare professionals, child, and caregivers—concurrent injections into the submandibular and parotid glands may be used in a subsequent BoNT‐A treatment session, diminishing both resting and stimulated saliva production.^9^

As with any treatment, side effects may occur after injections with BoNT‐A, specifically contributing to negative effects on oral motor function. The presumed underlying causes are associated with alterations in saliva viscosity, diffusion of BoNT‐A beyond the salivary gland (i.e. leading to muscle weakness), and BoNT‐A being overly effective (i.e. leading to xerostomia).^1^, ^10^, ^11^ Nevertheless, research on negative effects after BoNT‐A injections is relatively limited, with a predominant focus on treatment effectiveness. Studies that specifically report on safety tend to focus primarily on adverse events rather than a broader assessment of the included children.^12^ A prior study of our saliva control team assessed negative effects on oral motor function in a cohort of 209 children treated with submandibular injections.^13^ Negative effects occurred in 33%, with the majority being moderate and resolving spontaneously within 4 weeks. A study of 26 children treated with BoNT‐A in both the submandibular and parotid glands reported overall worsening of eating and drinking in 2 out of 20 (10%) children,^14^ although this rate should be interpreted cautiously given the small sample size.

We hypothesize that, given the functional role of the parotid gland, negative effects related to eating and swallowing might be more common for concurrent submandibular and parotid injections and that problems related to an excessively dry mouth may occur more frequently considering the injection of four major salivary glands. Within this vulnerable patient population, a carefully tailored treatment approach is essential. Should data suggest an elevated rate of these negative effects, adjustments in parental education before treatment are necessary and a reassessment of our treatment strategy becomes imperative. Therefore, this study aims to evaluate negative effects on oral motor function after concurrent BoNT‐A injections into the submandibular and parotid salivary glands.

## METHOD

A retrospective cohort study was conducted, using data from children (aged 4–18 years) with a developmental disability, primarily neurodevelopmental, who were treated with concurrent submandibular and parotid BoNT‐A injections to diminish drooling at the Radboudumc Amalia Children's Hospital between April 2004 and February 2023. Children frequently underwent multiple rounds of BoNT‐A injections as part of the treatment approach for drooling during their childhood; however, this study includes only the first concurrent submandibular and parotid injection per child, with the numbers representing unique individuals. Exclusion criteria were: (1) simultaneous use of systemic anticholinergic medication (i.e. between the date of injection and 32‐week follow‐up); (2) previous surgical saliva control treatment (e.g. submandibular duct relocation, submandibular gland excision, salivary duct ligation); and (3) no standard 8‐week follow‐up information available.

All included children received the injections as part of routine clinical care, involving an ultrasound‐guided injection of BoNT‐A (Botox, Allergan, Nieuwegein, the Netherlands) administered in the submandibular glands (bilaterally) and parotid glands (either unilaterally or bilaterally), while under general anaesthesia.^15^ Generally, a dose of 25 U BoNT‐A was used. Since our team applies a stepped care approach—starting with bilateral submandibular injections and opting for these concurrent submandibular and parotid injections only if the initial treatment is insufficient (with a few exceptions)—most children included in the study were not naive to BoNT‐A treatment.

Standard follow‐up appointments with a speech‐language therapist from our team were scheduled at 8 weeks and 32 weeks post‐injection, with the 8‐week follow‐up increasingly conducted via telephone in recent years. Three speech‐language therapists are part of our saliva control team, one of whom has been involved since the start of data collection. Both speech‐language therapists that joined the team later were familiarized with the assessment procedures to ensure consistency in measurement.

The local research ethics committee of the Radboudumc deemed the study exempt from ethical review (reference: 2023–16435). As this study comprised an analysis of data collected for routine clinical care, the committee waived the need to obtain informed consent for children no longer receiving active care (i.e. last visit to the outpatient clinic more than 7 years ago and/or deceased). For the remaining children, informed consent was obtained from parents and/or caregivers.

### Outcome definitions

#### Negative effects on oral motor function

As part of routine clinical care, the occurrence of negative effects was discussed during follow‐up evaluations with the speech‐language therapist at 8 weeks and 32 weeks post‐injection. Parents were specifically asked for any signs of probable negative effects or changes in the child's health condition. In addition, parents were encouraged to contact the saliva control team in case of any changes in oral motor function during the first 8 weeks. Clinical notes with details on these follow‐up evaluations and any contacts with parents apart from these evaluations were extracted from the electronic medical records of included children to evaluate the frequency of negative effects.

Negative effects were classified into one of five domains using the International Classification of Functioning, Disability and Health, Children and Youth version, in line with the classification in a previous study by our saliva control team:^13^, ^16^ (1) saliva swallowing (e.g. changes in saliva viscosity, increased choking on saliva, and/or discomfort during swallowing saliva); (2) eating (e.g. discomfort during eating [including coughing, gagging], deteriorated feeding pattern); (3) drinking (e.g. discomfort during drinking [including coughing, choking, dyspnoea]); (4) articulation (e.g. deteriorated speech); and (5) miscellaneous (e.g. sore throat, excessively dry mouth/lips, teeth grinding).

Subsequently, the severity of negative effects was categorized into one of three categories: (1) mild problems, comprising short‐term, transient changes in oral motor function, resulting in a slight impairment in swallowing, eating, drinking, and/or speaking, not leading to changes in lifestyle or doctor visits; (2) moderate problems, comprising transient changes in oral motor function, resulting in a moderate impairment in swallowing, eating, drinking, or speaking, requiring consultation by a general practitioner, leading to weight loss, or leading to changes in lifestyle; and (3) severe problems, comprising changes in oral motor function that require one or more days of hospitalization or substantial changes in feeding (e.g. tube feeding).

The frequency, severity, timing, and duration of negative effects were compared to a reference cohort from a previous study by our saliva control team, consisting of 209 children treated with BoNT‐A injections into the submandibular salivary glands (bilaterally).^13^

#### Treatment effect

At baseline and during follow‐up, the severity of drooling was evaluated semi‐objectively using the drooling quotient.^17^ The drooling quotient indicates the number of drooling episodes observed during a 5‐minute period while the child is active. Additionally, parents scored drooling severity using a Visual Analogue Scale (VAS) or Verbal Numerical Rating Scale (vNRS) ranging from 0 to 10 (i.e. no drooling to very severe drooling) and reported the Drooling Severity and Frequency Scale (DSFS). The DSFS evaluates the severity of drooling on a 5‐point scale (i.e. none to profuse) and the frequency of drooling on a 4‐point scale (i.e. never to constant).^18^ To define a clinically relevant treatment response, two different definitions based on changes in drooling quotient, VAS/vNRS, and DSFS at 8‐weeks post‐injection relative to baseline were used. For the first definition, children with at least a 50% reduction in drooling quotient and/or VAS/vNRS were classified as responders.^19^ For the second definition, responders were those who showed a 2‐point reduction in DSFS, encompassing at least a 1‐point reduction in both the severity and frequency component of the score.^20^

### Statistical analysis

Data processing and all statistical analyses were conducted using IBM SPSS Statistics (Version 29.0; IBM Corp., Armonk, NY, USA). Clinical characteristics and negative effects on oral motor function were analysed and summarized using descriptive statistics. Relative risks and 95% confidence intervals (CIs) were calculated to assess differences in negative effects between the current cohort (i.e. children treated with concurrent submandibular and parotid injections) and the reference cohort (i.e. children treated with submandibular injections). Differences in the number of affected oral motor domains and the severity, timing, and duration of negative effects were evaluated using Fisher's or Fisher–Freeman–Halton exact tests, with two‐sided *p*‐values less than 0.05 considered statistically significant.

Sensitivity analyses were conducted to evaluate how the overall negative effects rate was influenced by: (1) the number of previous submandibular injections (0, 1, or 2+), using a Fisher–Freeman–Halton exact test; and (2) the number of parotid glands injected, restricting the sample to children who received bilateral parotid injections, for which the frequencies in the full and restricted sample were compared. Additionally, a post hoc analysis explored differences in negative effects between children treated in the first (April 2004–December 2013) and second (January 2014–February 2023) decade of the study period, as well as between responders and non‐responders, with relative risks and 95% CIs computed or Fisher's exact tests conducted for these comparisons, as appropriate. Furthermore, we explored variations in treatment response rates, restricting the analysis to children with preceding submandibular BoNT‐A injections or those with bilateral parotid injections.

## RESULTS

### Clinical and treatment characteristics

We identified 146 children who were treated with first‐time concurrent BoNT‐A injections in the submandibular glands (bilaterally) and parotid glands (either unilaterally or bilaterally) between April 2004 and February 2023, of whom 125 were included in the analyses (Figure S1). Baseline characteristics of the included children, as well as details regarding drooling and treatment, are presented in Table 1.

**TABLE 1:** Baseline characteristics of children treated with concurrent submandibular and parotid BoNT‐A injections (*n* = 125).

Demographic and clinical characteristics	*n* (%)
Sex, female	47 (37.6)
Age at injection, years:months, median (IQR)	7:7 (4:5)
Developmental age	
<4 years	100 (80.0)
4–6 years	14 (11.2)
≥6 years	11 (8.8)
Diagnosis	
Spastic CP	30 (24.0)
Spastic/dyskinetic CP	21 (17.4)
Dyskinetic CP	5 (4.1)
Bulbar CP	1 (0.8)
Other developmental disability^a^	68 (54.4)
Disease course, progressive	10 (8.0)
Epilepsy	
Absent	62 (49.6)
Controlled	45 (36.0)
Refractory	18 (14.4)
Ambulatory level,^b^ walking (*n* = 68)	43 (63.2)
GMFCS level^c^ (*n* = 57)	
I–III	23 (40.4)
IV–V	34 (59.6)
Dysarthria	
Very severe	28 (22.4)
Severe	15 (12.0)
Moderate	9 (7.2)
Mild	8 (6.4)
Minimal	1 (0.8)
No dysarthria	37 (29.6)
No active speech	27 (21.6)
Dysphagia, EDACS or DMSS level	
I–III	85 (68.0)
IV–V	39 (31.2)
Unable to classify	1 (0.8)
Feeding method	
Tube^d^	31 (24.8)
Tube and oral	6 (4.8)
Oral	88 (70.4)
**Drooling characteristics** ^e^	
Type of drooling	
Anterior	90 (72.0)
Posterior	1 (0.8)
Anterior and posterior	34 (27.2)
DQ, median (IQR) (*n* = 115)	25.0 (32.5)
VAS/vNRS, median (IQR) (*n* = 124)	8.0 (2.0)
Drooling severity (*n* = 119)	
Moderate	3 (2.5)
Severe	23 (19.3)
Profuse	93 (78.2)
Drooling frequency (*n* = 120)	
Occasional	7 (5.8)
Frequent	40 (33.3)
Constant	73 (60.8)
**Treatment characteristics**	
Number of prior submandibular BoNT‐A injections	
0	12 (9.6)
1	73 (58.4)
≥2	40 (32.0)
Injected salivary glands^f^	
Both submandibular glands and both parotid glands	119 (95.2)
Both submandibular glands and one parotid gland	6 (4.8)
Dosage of BoNT‐A, per gland	
<25 U	11 (8.8)
25 U	113 (90.4)
>25 U	1 (0.8)

*Note*: All characteristics are reported as *n (%)* unless otherwise indicated.

Abbreviations: BoNT‐A, botulinum neurotoxin A; CP, cerebral palsy; DMSS, Dysphagia Management Staging Scale (levels I–III, absent, mild, or moderate swallowing or feeding disorder; levels IV–V, severe to profound swallowing or feeding disorder); DQ, drooling quotient; EDACS, Eating and Drinking Ability Classification System (levels I–III, eats and drinks with no or few limitations to efficiency and/or safety; levels IV–V, eats and drinks with significant limitations to safety or unable to eat or drink safely); GMFCS, Gross Motor Function Classification System (levels I–III, ambulatory; levels IV–V, non‐ambulatory); IQR, interquartile range; U, unit; VAS, Visual Analogue Scale; vNRS, Verbal Numerical Rating Scale.

^a^ For example, neurogenetic disorders or syndromes, metabolic disorders, epileptic encephalopathy, or traumatic brain injury.

^b^ For children with developmental disabilities other than CP.

^c^ For children with CP.

^d^ Includes several children who could still take some food or liquid by mouth (i.e. minimal tastes for pleasure).

^e^ Several numbers differ from total *n* because of missing data in outcome measures for some included children.

^f^ Unilateral parotid injections were chosen for several children because of factors such as their eating/chewing habits, parotid flow rate, ensuring that sufficient saliva remains, or exercising caution when these were the first injections to be administered.

As expected, a substantial majority of children (90.4%) were treated with submandibular injections at least once before the current treatment (Table 1). In the remaining 12 children (9.6%), concurrent submandibular and parotid injections were chosen right away, generally because the parotid salivary glands were expected to play a significant role in the drooling problem (i.e. supported by serous salivary consistency and frequent mouthing behaviour). At times, this decision was reinforced by severe posterior drooling symptoms (*n* = 4), necessitating maximum reduction in saliva production. Consistent with the treatment protocol, injections mostly (90.4%) involved the administration of 25 units BoNT‐A per gland and were predominantly (95.2%) delivered in both submandibular glands and both parotid glands.

According to our primary definition (i.e. at least 50% reduction in drooling quotient and/or VAS/vNRS), 55.2% (69/124; information was unavailable for one child) of the included children showed a clinically relevant response to treatment, which was 36.0% (45/116; information was unavailable for nine children) according to the secondary definition (i.e. at least 1‐point reduction in both subscales of the DSFS). Treatment response rates did not differ when excluding children with unilateral parotid injections (i.e. one parotid and both submandibular glands injected) or children who were not initially treated with BoNT‐A injections in the submandibular glands only.

### Frequency and characteristics of negative effects on oral motor function

A total of 45 children (36.0%) were reported to have experienced negative effects after treatment, of whom 28 children (62.2%) encountered negative effects in one oral motor domain and 17 (37.8%) in two or more domains (Table 2). Eating problems were the most prevalent negative effects, observed in 29 children (64.4%), followed by saliva swallowing problems (26.7%), drinking problems (17.8%), and articulation problems (8.9%). Thirteen children (28.9%) experienced negative effects that could not be classified into one of the specific oral motor domains, including problems resulting from excessively dry mouth or lips, changes in the odour of saliva, allergic reactions, and thickened saliva which could not be coughed up. Demographic and clinical characteristics at baseline were compared between children with and without negative effects on oral motor function (Table S1), suggesting a higher prevalence of CP (57.8% vs 38.7%, *p* = 0.061), very severe dysarthria (35.6% vs 15.0%, *p* = 0.013), and severe dysphagia (40.9% vs 27.3%, *p* = 0.159) in the subgroup with negative effects.

**TABLE 2:** Frequency and characteristics of negative effects after BoNT‐A injections.

	Current cohort (*n* = 125)	Reference cohort (*n* = 209)^a^	RR (95% CI) or *p* ^b^
**Reported negative effects**			
Yes	45 (36.0)	69 (33.0)	1.09 (0.81–1.48)
No	80 (65.0)	140 (67.0)	
**Characteristics of negative effects**	*n* = 45	*n* = 69	
Affected oral motor domain(s)^c^			
Saliva swallowing	12 (26.7)	22 (31.9)	0.83 (0.46–1.52)
Eating	29 (64.4)	51 (73.9)	0.87 (0.67–1.13)
Drinking	8 (17.8)	22 (31.9)	0.56 (0.27–1.14)
Articulation	4 (8.9)	4 (5.8)	1.53 (0.40–5.82)
Miscellaneous	13 (28.9)	15 (21.7)	1.33 (0.70–2.52)
Number of simultaneously affected domains			
1	28 (62.2)	37 (53.6)	0.725
2	14 (31.1)	23 (33.3)	
3	2 (4.4)	5 (7.2)	
4	1 (2.2)	4 (5.8)	
Severity^d^			
Mild	28 (62.2)	55 (79.7)	0.001
Moderate	15 (33.3)	7 (10.1)	
Severe	0 (0.0)	6 (8.7)	
*Not specified*	*2 (4.4)*	*1 (1.5)*	
Onset			
<1‐week post‐injection	25 (55.6)	54 (78.3)	0.059
1–8 weeks post‐injection	8 (17.8)	5 (7.2)	
*Not specified*	*12 (26.7)*	*10 (14.5)*	
Duration			
<1 week	2 (4.4)	12 (17.4)	0.111
1–4 weeks	22 (48.9)	25 (36.2)	
4–8 weeks	4 (8.9)	6 (8.7)	
8–32 weeks	1 (2.2)	7 (10.2)	
>32 weeks	1 (2.2)	2 (2.9)	
*Not specified*	*15 (33.3)*	*17 (24.6)*	
Interventions			
None reported	36 (80.0)	50 (72.5)	0.227
Telephone consultation	8 (17.8)	11 (15.9)	
Additional outpatient visit	0 (0.0)	3 (4.3)	
Hospital admission	0 (0.0)	4 (5.8)	
*Not specified*	*1 (2.2)*	*0 (0.0)*	
Advised management			
None reported	34 (75.6)	50 (72.5)	0.073
Start (or increase) of tube feeding	0 (0.0)	2 (2.9)	
Feeding/food consistency modifications	2 (4.4)	9 (13.0)	
Medication (i.e. mucolytics)	5 (11.1)	1 (1.5)	
Other	2 (4.4)	5 (7.2)	
*Not specified*	*2 (4.4)*	*2 (2.9)*	

*Note*: All characteristics are reported as *n* (%) unless otherwise stated.

Abbreviations: BoNT‐A, botulinum neurotoxin A; CI, confidence interval; RR, relative risks.

^a^ Data from the reference cohort, which consists of children treated with bilateral submandibular injections, were previously reported in a study conducted by our saliva control team.^13^

^b^ Relative risks and 95% confidence intervals are reported for binary outcomes, whereas *p*‐values from Fisher's or Fisher–Freeman–Halton exact tests are reported for differences in categorical distributions, as appropriate.

^c^ Saliva swallowing, including changes in saliva viscosity, increased choking on saliva, discomfort during saliva swallow; Eating, including discomfort (e.g. coughing, gagging) during eating, deteriorated feeding pattern; Drinking, including discomfort (e.g. coughing, choking, dyspnoea) during drinking; Articulation, including deteriorated speech; Miscellaneous, including sore throat, dry mouth, dry lips, teeth grinding. Categories are not mutually exclusive.

^d^ Mild includes short‐term, transient changes in oral motor function, not leading to changes in lifestyle or doctor visits. Moderate includes transient changes in oral motor function, leading to changes in lifestyle or requiring consultation by a general practitioner. Severe includes changes in oral motor function requiring one or more days of hospitalization or substantial changes in feeding (e.g. tube feeding).

Of the children who experienced negative effects, 40 had previously received submandibular BoNT‐A injections at our clinic, with six (15.0%) also experiencing (mild) negative effects on oral motor function after those earlier injections.

Negative effects were generally classified as mild (62.2%) or moderate (33.3%), with no instances of severe negative effects reported (Table 2). Most of the problems (55.6%) emerged within the first week post‐injection and a majority (53.3%) had resolved within 4 weeks. Two children (4.4%) experienced negative effects lasting more than 8 weeks; one persisted for 12 weeks, while the other had not resolved by 32‐weeks post‐injection. In eight cases (17.8%) an additional telephone consultation was required. In 11 cases (24.4%), certain approaches to manage negative effects were advised, including recommendations to modify feeding practices, adapt food consistency, start mucolytic medication, use a tongue cleaner, provide sips of water, or use an antibacterial mouthwash in eight children, while the specific management strategy for two of these cases was not further specified.

No differences were observed in the overall negative effects rate when excluding children with unilateral parotid injections (35.3% compared to 36.0% in the entire cohort). Similarly, there were no notable differences when comparing children treated before and after 1st January 2014 (36.7% vs 34.8%; relative risks 1.06; 95% CI 0.65–1.72), or when comparing responders to non‐responders (34.8% vs 38.2%; relative risks 0.91; 95% CI 0.57–1.45). However, Fisher's exact test indicated a difference in the severity of negative effects between responders and non‐responders (*p* = 0.023), with responders experiencing moderately severe negative effects more frequently than non‐responders (50% vs 14%) (Table S2). Additionally, negative effects appeared to be more commonly reported among children with a single previous submandibular injection (44.4%), compared to those with two or more previous injections (27.5%) and those without any prior injections (16.7%) (*p* = 0.076), although this should be interpreted with caution considering the small size of these subgroups.

### Comparison to a reference cohort treated with submandibular injections

The overall negative effects rate in the current cohort, consisting of children treated with concurrent submandibular and parotid injections, was similar to the reference cohort, consisting of children treated with submandibular injections (36.0% vs 33.0%; relative risks 1.09; 95% CI 0.80–1.48), as shown in Table 2. Additionally, the distribution of negative effects across three oral motor domains (i.e. eating, saliva swallowing, articulation) was comparable, although drinking problems appeared to be reported slightly less often in the current cohort (relative risks 0.56; 95% CI 0.27–1.14).

There was a notable shift in the classified severity of negative effects when compared to the reference cohort (*p* = 0.001) (Table 2). Specifically, a greater proportion of children in the current cohort encountered moderate negative effects (33.3% vs 10.1%). Interestingly, while the reference cohort exhibited several cases of severe negative effects (8.7%), no severe negative effects were observed in the current cohort. Additionally, the percentage of children experiencing negative effects across multiple domains appeared somewhat reduced in the current cohort (37.8% vs 46.4%), although the difference in overall distribution was not statistically significant (*p* = 0.725).

In contrast to the reference cohort, no additional outpatient visits or hospital admissions were required in children included in the current study, nor did any child require initiation or increase of tube feeding (Table 2). However, the advice to start mucolytic medications to manage thick saliva was given more often (11.1% vs 1.5%).

## DISCUSSION

To our knowledge, this study describes the first comprehensive examination of negative effects on oral motor function among children treated with concurrent submandibular and parotid gland injections within a cohort of this size. Negative effects were reported for one‐third of the included children, primarily manifesting as eating‐related problems and difficulties in saliva swallowing. Encouragingly, the majority of these problems were classified as mild and resolved within 4‐weeks post‐injection, with only two cases lasting longer than 8 weeks.

The overall reported rate for any negative effects after concurrent submandibular and parotid BoNT‐A injections differs considerably across published literature, generally ranging from 0% to 40% and occasionally reaching up to 78%.^21^ Nevertheless, it is important to note that differences in treatment protocol and outcome definitions (e.g. adverse events, side effects, complications, secondary effects) may have influenced these rates, along with differences in characteristics of the study population.^21^ For example, Berweck et al. recently reported adverse events in 27 cases (18.2%), but these mostly consisted of non‐treatment‐related respiratory infections.^22^ Our focus was on expected negative effects of treatment, and it is not always evident whether these are encompassed in the analyses of other studies.

Generally, the negative effects rate in the current study mirrored our reference cohort treated with submandibular injections. However, these cohorts differed in several distinctive clinical characteristics, particularly in the proportion of children with CP, a previously identified risk factor for negative effects.^13^ Moreover, a majority of included children had prior exposure to BoNT‐A in the submandibular glands because of our stepped care approach, and a step‐up to concurrent submandibular and parotid injections will likely not have been indicated in those with severe negative effects to initial injections. It remains uncertain whether this may have impacted susceptibility to negative effects in the current cohort. Additionally, it is worth considering that parents with prior experience with this treatment may have taken additional precautions during this treatment session that we were unaware of, got used to certain side effects, or were less worried, further influencing outcomes. These considerations indicate that the negative effect profile of concurrent injections in children previously treated with submandibular injections should not simply be assumed to mirror that of stand‐alone concurrent injections. Establishing the generalizability of our findings in a treatment‐naive cohort is therefore crucial.

Although negative effects primarily manifested as problems with eating, the prevalence of eating‐related problems did not appear to differ from our reference cohort, in contrast to our hypothesis. However, differences in feeding methods between the two cohorts should be taken into account, with 70.4% primarily fed orally in the current cohort compared to 83.7% in the reference cohort, meaning that the population of children at risk for negative effects during mealtimes was smaller. Reid et al. included a population of children with a higher prevalence of oral feeding (84.6%), similar to our reference cohort, and found worsening of eating and drinking in only 10% of children.^14^ However, this lower proportion may be attributed to factors including the relatively small sample size or the method of assessing secondary effects, based on a questionnaire at 4‐weeks post‐treatment. A certain correlation between treatment effect and the occurrence of negative effects was hypothesized before the study, associated either with technical failure of the injections (i.e. reflected by treatment non‐response) or with excessive effectiveness of the treatment and/or the thickening of saliva^10^ (i.e. reflected by treatment response). Although no difference in the overall negative effects rate could be identified, children classified as responders more often appeared to have moderately severe negative effects rather than mild negative effects compared to non‐responders, potentially reflecting this second hypothesis.

Interestingly, no severe cases of negative effects were found in the current cohort, a notable contrast to the reference cohort as well as findings in existing literature. For example, Khan et al. reported adverse events in 15 children (33.3%), with five experiencing severe complications.^23^ Similarly, a study by Chan et al. revealed adverse events in 19 children (15.8%), with major complications in five cases.^12^ This discrepancy may be a testament to the value of our stepped care approach, as children experiencing severe negative effects after submandibular BoNT‐A injections are unlikely to receive concurrent submandibular and parotid injections. Analogous to drug titration, this approach may enable the identification of those susceptible to severe side effects at a lower BoNT‐A dose (i.e. submandibular injections), potentially preventing more severe negative effects that might arise when submandibular and parotid injections would be given. However, it should be acknowledged that this discrepancy could also be a consequence of prior exposure to BoNT‐A in general, rather than a distinct difference between submandibular and concurrent submandibular and parotid injections.

To better inform decision‐making, it is crucial to understand both the expected treatment effects and potential side effects of concurrent BoNT‐A injections for drooling. Nevertheless, it is important to recognize that response rates can vary depending on the criteria used and may not fully reflect individual satisfaction or perceived effectiveness.^24^ Likewise, negative effects may not always be perceived as outweighing the benefits of treatment but should rather be considered as important aspects to be anticipated. Therefore, our study aimed to provide caregivers with realistic expectations about potential negative effects and their severity, ensuring they are well informed before treatment and allowing them to make necessary adaptations to support the child if changes in oral motor function occur. Moreover, it remains important to apply an individualized treatment approach, in which the benefits and potential negative effects of treatment are continuously weighed for each individual child.

The strength of our study lies in the large sample size and comprehensive collection of information regarding negative effects, facilitated by routine follow‐up evaluations where documenting any negative effects is standard practice. However, several limitations should be acknowledged. First, mild negative effects may not have been consistently disclosed by parents during follow‐up evaluations or may not have been deemed substantial enough to be documented in the medical records. Second, it has been hypothesized that negative effects may occur more frequently in children exposed to multilevel BoNT‐A injections, where intraglandular injections are combined with injections into the skeletal muscles.^13^ We were unable to explore this in the current cohort. Third, the data for this study were collected over a 20‐year period, involving three speech‐language therapists in follow‐up. However, a thorough onboarding process was implemented to standardize follow‐up procedures, thereby maintaining consistency and minimizing variations in reporting negative effects after BoNT‐A injections.

In conclusion, this study sheds light on the frequency, severity, and duration of negative effects on oral motor function after concurrent submandibular and parotid gland BoNT‐A injections, in children predominantly treated with submandibular injections initially, providing valuable insights to inform parents and children before treatment. Our findings suggest that clinicians can confidently consider adding parotid injections to the treatment protocol when submandibular injections prove ineffective, given the comparable negative effect profile in this specific setting. While careful interpretation of these results is warranted, considering that most children were previously exposed to intraglandular BoNT‐A, our findings also prompt the hypothesis that a stepped care approach may be beneficial. Specifically, it may prevent severe negative effects after concurrent submandibular and parotid injections by selectively excluding children at risk for these consequences at an earlier stage. Further investigation is needed to elucidate the nuances in this observed pattern.

## Supporting information


**Table S1:** Patient characteristics of children treated with concurrent submandibular and parotid BoNT‐A injections stratified for occurrence of negative effects.

**Table S2:** Frequency and characteristics of negative effects after BoNT‐A injections stratified for treatment response.

**Figure S1:** Study population flowchart.

## Data Availability

The data that support the findings of this study are available on request from the corresponding author. The data are not publicly available as participants of this study did not give written consent for this.

## References

[1] Reddihough DS , Erasmus CE , Johnson H , McKellar GM , Jongerius PH . Botulinum toxin assessment, intervention and aftercare for paediatric and adult drooling: international consensus statement. European Journal of Neurology. 2010;17 Suppl 2:109–21. 20633182 10.1111/j.1468-1331.2010.03131.x

[2] Speyer R , Cordier R , Kim JH , Cocks N , Michou E , Wilkes‐Gillan S . Prevalence of drooling, swallowing, and feeding problems in cerebral palsy across the lifespan: a systematic review and meta‐analyses. Developmental Medicine & Child Neurology. 2019;61(11):1249–58. 31328797 10.1111/dmcn.14316

[3] van der Burg JJW , Jongerius PH , van Limbeek J , van Hulst K , Rotteveel JJ . Drooling in children with cerebral palsy: a qualitative method to evaluate parental perceptions of its impact on daily life, social interaction, and self‐esteem. International Journal of Rehabilitation Research. 2006;29(2):179–82. 16609333 10.1097/01.mrr.0000194395.64396.f1

[4] van der Burg JJW , Jongerius PH , van Hulst K , van Limbeek J , Rotteveel JJ . Drooling in children with cerebral palsy: effect of salivary flow reduction on daily life and care. Developmental Medicine & Child Neurology. 2006;48(2):103–7. 16417664 10.1017/S0012162206000235

[5] Delsing CP , Bekkers S , Erasmus CE , van Hulst K , van den Hoogen FJ . Posterior drooling in children with cerebral palsy and other neurodevelopmental disorders. Developmental Medicine & Child Neurology. 2021. 10.1111/dmcn.14888 33844298

[6] Heikel T , Patel S , Ziai K , Shah SJ , Lighthall JG . Botulinum Toxin A in the Management of Pediatric Sialorrhea: A Systematic Review. Annals of Otology, Rhinology, and Laryngology. 2023;132(2):200–6. 35176902 10.1177/00034894221078365 PMC9834812

[7] Glader L , Delsing C , Hughes A , Parr J , Pennington L , Reddihough D , et al. AACPDM Care Pathway for sialorrhea in Cerebral Palsy 2017 [updated June 2018]. Available from: http://www.aacpdm.org/publications/care‐pathways/sialorrhea.

[8] Erasmus CE , Van Hulst K , Rotteveel LJ , Jongerius PH , Van Den Hoogen FJ , Roeleveld N , Rotteveel JJ . Drooling in cerebral palsy: hypersalivation or dysfunctional oral motor control? Developmental Medicine & Child Neurology. 2009;51(6):454–9. 19207297 10.1111/j.1469-8749.2008.03243.x

[9] Erasmus CE , van Hulst K , Rotteveel JJ , Willemsen MAAP , Jongerius PH . Clinical practice: swallowing problems in cerebral palsy. European Journal of Pediatrics. 2012;171(3):409–14. 21932013 10.1007/s00431-011-1570-y PMC3284655

[10] Erasmus CE , van Hulst K , van den Hoogen FJA , van Limbeek J , Roeleveld N , Veerman EC , et al. Thickened saliva after effective management of drooling with botulinum toxin A. Developmental Medicine & Child Neurology. 2010;52(6):e114–e8. 20163435 10.1111/j.1469-8749.2009.03601.x

[11] Nordgarden H , Osterhus I , Moystad A , Asten P , Johnsen UL , Storhaug K , Loven JO . Drooling: are botulinum toxin injections into the major salivary glands a good treatment option? Journal of Child Neurology. 2012;27(4):458–64. 21940695 10.1177/0883073811419365

[12] Chan KH , Liang C , Wilson P , Higgins D , Allen GC . Long‐term safety and efficacy data on botulinum toxin type A: an injection for sialorrhea. JAMA Otolaryngology ‐ Head & Neck Surgery. 2013;139(2):134–8. 23429943 10.1001/jamaoto.2013.1328

[13] van Hulst K , Kouwenberg CV , Jongerius PH , Feuth T , van den Hoogen FJ , Geurts AC , Erasmus CE . Negative effects of submandibular botulinum neurotoxin A injections on oral motor function in children with drooling due to central nervous system disorders. Developmental Medicine & Child Neurology. 2017;59(5):531–7. 27901263 10.1111/dmcn.13333

[14] Reid SM , Walstab JE , Chong D , Westbury C , Reddihough DS . Secondary effects of botulinum toxin injections into salivary glands for the management of pediatric drooling. Journal of Craniofacial Surgery. 2013;24(1):28–33. 23348253 10.1097/SCS.0b013e31827102a0

[15] Scheffer AR , Erasmus C , van Hulst K , van Limbeek J , Jongerius PH , van den Hoogen FJ . Efficacy and duration of botulinum toxin treatment for drooling in 131 children. Archives of Otolaryngology ‐ Head & Neck Surgery. 2010;136(9):873–7. 20855679 10.1001/archoto.2010.147

[16] World Health Organization . International classification of functioning, disability and health: children and youth version: ICF‐CY. World Health Organization 2007.

[17] van Hulst K , Lindeboom R , van der Burg J , Jongerius P . Accurate assessment of drooling severity with the 5‐minute drooling quotient in children with developmental disabilities. Developmental Medicine & Child Neurology. 2012;54(12):1121–6. 23094939 10.1111/j.1469-8749.2012.04428.x

[18] Thomas‐Stonell N , Greenberg J . Three treatment approaches and clinical factors in the reduction of drooling. Dysphagia. 1988;3(2):73–8. 3271655 10.1007/BF02412423

[19] Bekkers S , Delsing CP , Kok SE , van Hulst K , Erasmus CE , Scheffer ART , van den Hoogen FJA . Randomized controlled trial comparing botulinum vs surgery for drooling in neurodisabilities. Neurology. 2019;92(11):e1195–e204. 30728311 10.1212/WNL.0000000000007081

[20] National Guideline Alliance UK . Cerebral palsy in under 25s: assessment and management. 2017. 28151611

[21] Orriëns LB , van Hulst K , van der Burg JJW , van den Hoogen FJA , Willemsen M , Erasmus CE . Comparing the evidence for botulinum neurotoxin injections in paediatric anterior drooling: a scoping review. European Journal of Pediatrics. 2023. 10.1007/s00431-023-05309-1 PMC10858158 37924348

[22] Berweck S , Bonikowski M , Kim H , Althaus M , Flatau‐Baque B , Mueller D , Banach MD . Placebo‐controlled clinical trial of incobotulinumtoxinA for sialorrhea in children: SIPEXI. Neurology. 2021;02. 10.1212/WNL.0000000000012573 PMC8520391 34341153

[23] Khan WU , Campisi P , Nadarajah S , Shakur YA , Khan N , Semenuk D , et al. Botulinum toxin A for treatment of sialorrhea in children: An effective, minimally invasive approach. Archives of Otolaryngology ‐ Head & Neck Surgery. 2011;137(4):339–44. 21242533 10.1001/archoto.2010.240

[24] van Hulst K , van der Burg JJW , Jongerius PH , Geurts ACH , Erasmus CE . Changes in severity and impact of drooling after submandibular gland botulinum neurotoxin A injections in children with neurodevelopmental disabilities. Developmental Medicine & Child Neurology. 2020;62(3):354–62. 31729034 10.1111/dmcn.14391 PMC7028146

