# Supplementary material for: Negative effects on oral motor function after submandibular and parotid botulinum neurotoxin A injections for drooling in children with developmental disabilities
Source: Dev Med Child Neurol. 2024 Oct 24;67(5):656–64. doi: 10.1111/dmcn.16131 (PMC11965966; doi:10.1111/dmcn.16131)
Supplement: Supplementary file 1 — Table S1: Patient characteristics of children treated with concurrent submandibular and parotid BoNT‐A injections stratified for occurrence of negative effects. [file DMCN-67-656-s002.pdf]

Supplementary Table 1. Patient characteristics of children treated with concurrent submandibular and parotid BoNT-A injections stratified for occurrence of negative effects

	No negative effects (n = 80)		Negative effects (n = 45)	
**Sex**, female	32	(40.0)	15	(33.3)
**Age at injection**, years and months, *median (IQR)*	8y 3m	(4y 8m)	7y 3m	(2y 11m)
**Developmental age**				
<4 years	65	(81.3)	35	(77.8)
4-6 years	9	(11.3)	5	(11.1)
≥6 years	6	(7.5)	5	(11.1)
**Diagnosis**				
Spastic CP	16	(20.0)	14	(31.1)
Spastic/dyskinetic CP	12	(15.0)	9	(20.0)
Dyskinetic CP	2	(2.5)	3	(6.7)
Bulbar CP	1	(1.3)	0	(0.0)
Other neurodevelopmental disability [a]	49	(61.3)	19	(42.2)
**Disease course**, progressive	6	(7.5)	4	(8.9)
**Epilepsy**				
Absent	36	(45.0)	26	(57.8)
Controlled	31	(38.8)	14	(31.1)
Refractory	13	(16.3)	5	(11.1)
**Ambulatory level** [b], walking	42	(52.5)	23	(51.1)
**Dysarthria**				
Very severe	12	(15.0)	16	(35.6)
Severe	10	(12.5)	5	(11.1)
Moderate	5	(6.3)	4	(8.9)
Mild	4	(5.0)	4	(8.9)
Minimal	1	(1.3)	0	(0.0)
No dysarthria	26	(32.5)	11	(24.4)
No active speech	22	(27.5)	5	(11.1)
**Dysphagia**, EDACS or DMSS level				
I-III	58	(72.5)	27	(60.0)
IV-V	21	(26.3)	18	(40.0)
Unable to classify	1	(1.3)	0	(0.0)
**Feeding method**				
Tube [c]	17	(21.3)	14	(31.1)
Tube and oral	5	(6.3)	1	(2.2)
Oral	58	(72.5)	30	(66.7)
**Type of drooling**				
Anterior	58	(72.5)	32	(71.1)
Posterior	1	(1.3)	0	(0.0)
Anterior and posterior	21	(26.3)	13	(28.9)
**Number of prior submandibular BoNT-A injections**				
0	10	(12.5)	2	(4.4)
1	41	(51.2)	32	(71.1)
≥2	29	(36.3)	11	(24.4)

All characteristics are reported as *n (%)* unless otherwise indicated.

[a] e.g. neurogenetic disorders or syndromes, metabolic disorders, epileptic encephalopathy, or traumatic brain injury.

[b] GMFCS level I-III for children with CP.

[c] Includes several children who could still take some food or liquid by mouth (i.e. minimal tastes for pleasure)

Abbreviations: CP, cerebral palsy; DMSS, dysphagia management staging scale (levels I–III, absent, mild, or moderate swallowing or feeding disorder; levels IV–V, severe to profound swallowing or feeding disorder); EDACS, eating and drinking ability classification system (levels I–III, eats and drinks with no or few limitations to efficiency and/or safety; levels IV–V, eats and drinks with significant limitations to safety or unable to eat or drink safely).
