# Supplementary material for: Negative effects on oral motor function after submandibular and parotid botulinum neurotoxin A injections for drooling in children with developmental disabilities
Source: Dev Med Child Neurol. 2024 Oct 24;67(5):656–64. doi: 10.1111/dmcn.16131 (PMC11965966; doi:10.1111/dmcn.16131)
Supplement: Supplementary file 2 — Table S2: Frequency and characteristics of negative effects after BoNT‐A injections stratified for treatment response. [file DMCN-67-656-s001.pdf]

Supplementary Table 2. Frequency and characteristics of negative effects following BoNT-A injections stratified for treatment response

	Responders [a] (n = 69)		Non-responders [a] (n = 55)		RR (95% CI) or *p* value [b]
**Reported negative effects**	n	(%)	n	(%)	
Yes	24	(34.8)	21	(38.2)	0.91 (0.57-1.45)
No	45	(65.2)	34	(61.8)	
**Characteristics of negative effects**	*n = 24*		*n = 21*		
**Affected oral motor domain(s)** [c]					
Saliva swallowing	6	(25.0)	6	(28.6)	0.88 (0.33-2.30)
Eating	17	(70.8)	12	(57.1)	1.24 (0.79-1.95)
Drinking	4	(16.7)	4	(19.0)	0.88 (0.25-3.07)
Articulation	1	(4.2)	3	(14.3)	0.29 (0.03-2.60)
Miscellaneous	8	(33.3)	5	(23.8)	1.40 (0.54-3.63)
**Number of simultaneously affected domains**					
1	13	(54.2)	15	(71.4)	.413
2	10	(41.7)	4	(19.0)	
3	1	(4.2)	1	(4.8)	
4	0	(0.0)	1	(4.8)	
**Severity** [d]					
Mild	11	(45.8)	17	(81.0)	.023
Moderate	12	(50.0)	3	(14.3)	
Severe	0	(0.0)	0	(0.0)	
Not specified	*1*	*(4.2)*	*1*	*(4.8)*	
**Onset**					
<1 week post-injection	15	(62.5)	10	(47.6)	.695
1-8 weeks post-injection	4	(16.7)	4	(19.0)	
Not specified	*5*	*(20.8)*	*7*	*(33.3)*	
**Duration**					
<1 week	1	(4.2)	1	(4.8)	.240
1-4 weeks	12	(50.0)	10	(47.6)	
4-8 weeks	4	(16.7)	0	(0.0)	
8-32 weeks	0	(0.0)	1	(4.8)	
>32 weeks	1	(4.2)	0	(0.0)	
Not specified	*6*	*(25.0)*	*9*	*(42.9)*	

All characteristics are reported as n (%). [a] Clinically relevant response to treatment was defined as ≥50% reduction in drooling quotient and/or visual analogue scale/verbal numerical rating scale for drooling severity at the 8-week follow-up compared to baseline.

[b] Relative risks (RR) and 95% confidence intervals (95% CI) are reported for binary outcomes, whereas *p* values from Fisher's or Fisher-Freeman-Halton exact tests are reported for differences in categorical distributions, as appropriate.

[c] *Saliva swallowing,* including changes in saliva viscosity, increased choking on saliva, discomfort during saliva swallow; *Eating*, including discomfort (e.g., coughing, gagging) during eating, deteriorated feeding pattern; D*rinking*, including discomfort (e.g., coughing, choking, dyspnoea) during drinking; *Articulation*, including deteriorated speech; *Miscellaneous,* including sore throat, dry mouth, dry lips, teeth grinding. Categories are not mutually exclusive.

[d] *Mild* includes short-term, transient changes in oral motor function, not leading to changes in lifestyle or doctor visits. *Moderate* includes transient changes in oral motor function, leading to changes in lifestyle or requiring consultation by a general practitioner. *Severe* includes changes in oral motor function requiring one or more days of hospitalisation or substantial changes in feeding (e.g. tube feeding).
