# Supplementary figures and images for: Negative effects on oral motor function after submandibular and parotid botulinum neurotoxin A injections for drooling in children with developmental disabilities
Source: Dev Med Child Neurol. 2024 Oct 24;67(5):656–64. doi: 10.1111/dmcn.16131 (PMC11965966; doi:10.1111/dmcn.16131)

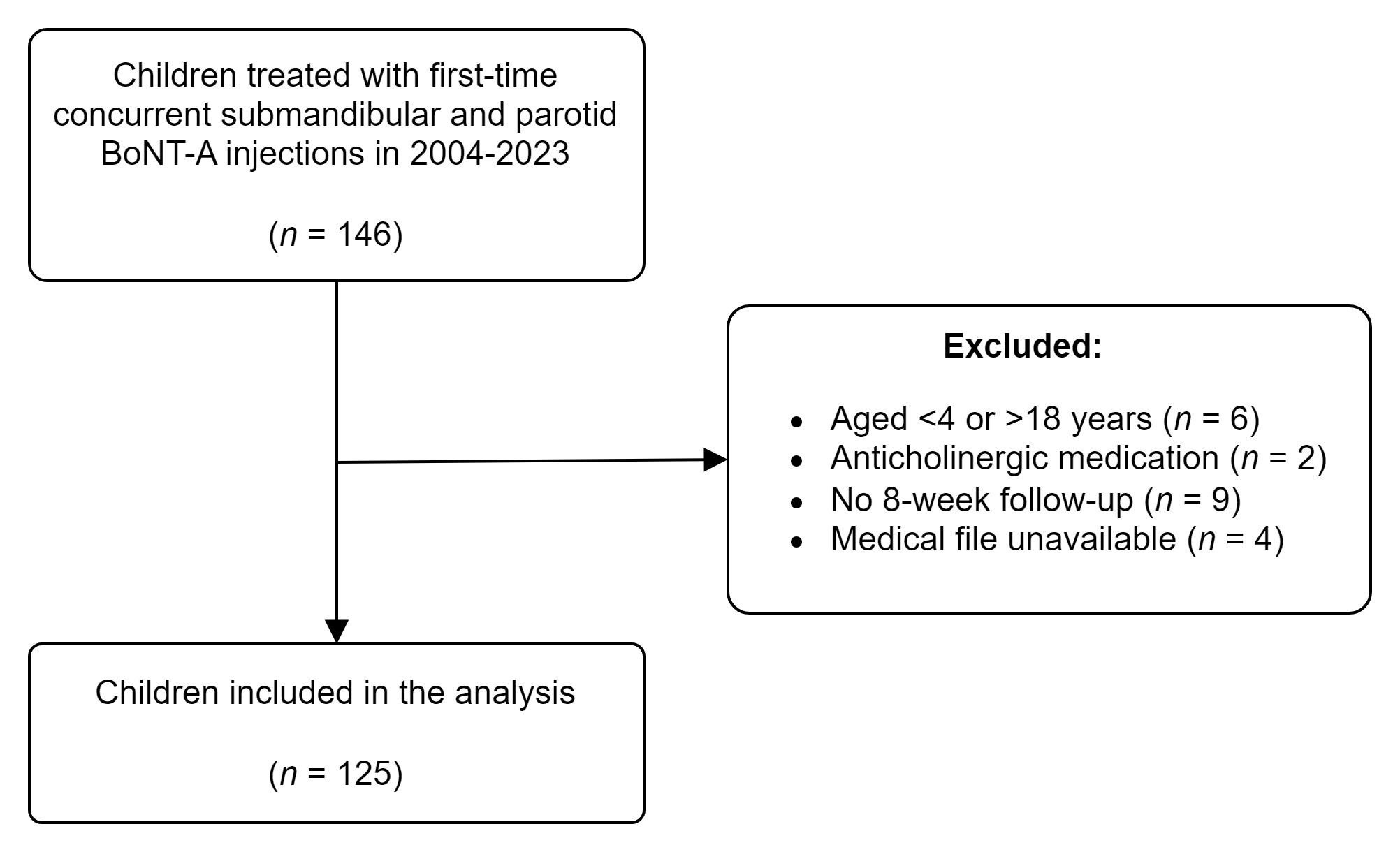

Supplement: Supplementary file 3 — Figure S1: Study population flowchart. [file DMCN-67-656-s003.jpg]
